# Effects of a Cognitive Training With and Without Additional Physical Activity in Healthy Older Adults: A Follow-Up 1 Year After a Randomized Controlled Trial

**DOI:** 10.3389/fnagi.2018.00407

**Published:** 2018-12-18

**Authors:** Elke Kalbe, Mandy Roheger, Kay Paluszak, Julia Meyer, Jutta Becker, Gereon R. Fink, Juraj Kukolja, Andreas Rahn, Florian Szabados, Brunhilde Wirth, Josef Kessler

**Affiliations:** ^1^Department of Medical Psychology, Neuropsychology and Gender Studies & Center for Neuropsychological Diagnostics and Intervention, University Hospital Cologne, Cologne, Germany; ^2^Institute for Interdisciplinary Dermatological Prevention and Rehabilitation, Osnabrück University, Osnabrück, Germany; ^3^Institute of Human Genetics, University Hospital Cologne, Cologne, Germany; ^4^Department of Neurology, University Hospital Cologne, Cologne, Germany; ^5^Cognitive Neuroscience, Institute of Neuroscience and Medicine (INM-3), Research Center Jülich, Jülich, Germany; ^6^Department of Neurology, HELIOS University Hospital Wuppertal, Wuppertal, Germany; ^7^Department of Geriatrics, St. Franziskus-Hospital Lohne, Lohne, Germany; ^8^Laboratory Services Laborarztpraxis Osnabrück, Osnabrück, Germany

**Keywords:** combined lifestyle intervention, predictor, neurobiological mechanisms, physical training, cognitive training, healthy older adults, follow-up, RCT

## Abstract

**Background:** Combining cognitive training (CT) with physical activity (CPT) has been suggested to be most effective in maintaining cognition in healthy older adults, but data are scarce and inconsistent regarding long-term effects (follow-up; FU) and predictors of success.

**Objective:** To investigate the 1-year FU effects of CPT versus CT and CPT plus counseling (CPT+C), and to identify predictors for CPT success at FU.

**Setting and Participants:** We included 55 healthy older participants in the data analyses; 18 participants (CPT group) were used for the predictor analysis.

**Interventions:** In a randomized controlled trial, participants conducted a CT, CPT, or CPT+C for 7 weeks.

**Outcome Measures:** Overall cognition, verbal, figural, and working memory, verbal fluency, attention, planning, and visuo-construction.

**Results:** While within-group comparisons showed cognitive improvements for all types of training, only one significant interaction *Group* × *Time* favoring CPT in comparison to CPT+C was found for overall cognition and verbal long-term memory. The most consistent predictor for CPT success (in verbal short-term memory, verbal fluency, attention) was an initial low baseline performance. Lower education predicted working memory gains. Higher levels of insulin-like growth factor 1 (IGF-1) and lower levels of brain-derived neurotrophic factor at baseline (BDNF) predicted alternating letter verbal fluency gains.

**Discussion:** Within-group comparisons indicate that all used training types are helpful to maintain cognition. The fact that cognitive and sociodemographic data as well as nerve growth factors predict long-term benefits of CPT contributes to the understanding of the mechanisms underlying training success and may ultimately help to adapt training to individual profiles.

**Clinical Trial Registration:** WHO ICTRP (http://apps.who.int/trialsearch/), identifier DRKS00005194.

## Introduction

Cognitive (Kelly et al., 2014a) as well as physical interventions (Kelly et al., 2014b) have the potential to enhance cognition in healthy older adults and thus constitute promising approaches to prevent cognitive aging or even to delay the onset of cognitive impairment or dementia (Smith, 2016).

Recently, it has been discussed whether the combination of cognitive training (CT) and physical training (PT) may yield stronger effects on cognition (e.g., Bamidis et al., 2014; Law et al., 2014). To date, however, data remain scarce and inconclusive. In a systematic review conducted by Rahe and Kalbe (2015), three out of four included controlled studies demonstrated a superiority of a combined training, while all four studies with a randomized controlled study design (RCT) failed to show an additional benefit of the combined training. However, the authors pointed out that the heterogeneity of the studies concerning, e.g., the intensity and frequency of the training and study designs, thus far limit any definitive conclusion. In a recent meta-analysis, Zhu et al. (2016) analyzed the effects of combined cognitive and physical interventions (CPT) on cognition in healthy older adults. This analysis, which included twenty controlled studies comprising 2667 participants, revealed a significant (albeit small) overall effect in favor of CPT versus (active or passive) control groups (standardized mean difference, SMD = 0.29). A recent systematic review of Joubert and Chainay (2018) stated that CT and PT both have positive outcomes for brain structure and function, and can improve cognition. Combined PT and CT showed an advantage compared to single trainings. However, the authors stated that data is far from being conclusive and that further studies are necessary to be able to draw robust conclusions in favor of combined training. A relevant gap of knowledge refers to the lack of follow-up (FU) data in the existing studies on CPT. Zhu et al. (2016) identified only three studies that analyzed the cognitive effects of CPT after 3 months (Linde and Alfermann, 2014), 1 year (Rahe et al., 2015c), and 5 years (Oswald et al., 2006), showing moderate effect sizes (SMD = 0.61) at time of FUs on global cognition when CPT was compared to passive (Oswald et al., 2006; Linde and Alfermann, 2014) and also active control groups (Rahe et al., 2015c). Notably, data were too heterogeneous and too few to analyze the effects on other cognitive domains. Taken together, data are promising concerning long-term effects of CPT on cognition (Zhu et al., 2016), but more research is necessary, especially as long-term effects are obviously the ultimate goal for interventions on cognition in healthy older adults.

In a recent RCT, we (Rahe et al., 2015a) studied the effects of CT, CPT, and CPT plus motivational physical activity counseling (CPT+C) on cognitive and physical fitness in healthy older adults. Results indicated that all types of interventions enhanced cognition as evidenced by gains in within-comparisons, while no evidence was found that CPT was superior to CT. However, there was a significant interaction effect Group × Time in favor of the CPT+C in comparison to the CPT in two executive tasks (alternating verbal fluency and a planning task). This effect was not assigned to additional PT gains, as physical fitness was more enhanced in the CPT group (as well as the brain-derived neurotrophic factor, BDNF, which plays a crucial role in brain plasticity, Valenzuela et al., 2007; Lista and Sorrentino, 2010). Instead it was suggested that counseling especially trained cognitive strategies and planning abilities. To the best knowledge of the authors, long-term effects of CPT+C in comparison to CT or CPT have not yet been studied.

One further aspect that is to date underinvestigated is the question which factors predict training success in healthy older adults. Any insights into this could help to adapt a training to individual profiles. Several inconclusive variables (e.g., sociodemographic variables, training performance, genetic variables and neurotrophic growth factors) have been identified that have predictive value for cognitive improvement in this group induced by cognitive interventions. For example, studies have reported that individuals with a low baseline performance benefitted more from CT (Langbaum et al., 2009; Whitlock et al., 2012; Zinke et al., 2014; Rahe et al., 2015a), although another study found the opposite result (Fairchild et al., 2013). Furthermore, participants with a higher self-rated health (Rebok et al., 2013), with younger age (Dorfman and Ager, 1989; Verhaeghen et al., 1992; Zinke et al., 2014), and a higher education (Langbaum et al., 2009) were found to benefit more from CT. However, data are inconclusive yet.

One possible further predictor of CT outcome is the apolipoprotein E (apoE 4) allele. A meta-analysis revealed that healthy older adults, who are carriers of the apoE4 allele, perform significantly worse on measures of episodic memory and overall global cognitive ability (Wisdom et al., 2011). Remarkably, a recent study showed that apoE4 can predict outcome in a cognitive stimulation program in patients with MCI, indicating that carrier of the apoE4 allele showed less improvement in memory than non-carriers (Binetti et al., 2013). In the above mentioned RCT of Rahe et al. (2015a), the apoE polymorphism predicted performance in an alternating letter verbal fluency task, indicating that non-carriers of the apoE 4 allele showed gains in executive functions compared to carriers of the apoE 4 allele. To the best of our knowledge, this study was the first that reported predictors of cognitive gains after CPT in healthy older adults.

As mentioned earlier, neurotrophic growth factors play a crucial role in brain plasticity (for a review see Valenzuela et al., 2007; Lista and Sorrentino, 2010). Especially BDNF, insulin-like growth factor 1 (IGF-1), and vascular endothelial growth factor (VEGF) have been studied as complementary indicators of exercise-induced neuro-, synapto-, and angio-genesis (for an overview see Cotman et al., 2007). Rahe et al. (2015a) showed that lower initial blood levels of BDNF predicted an improvement in alternating letter verbal fluency after CPT, showing that for this executive task BDNF level was essential for training-induced plasticity. First studies suggested an association between CT and changes in serum levels of BDNF (Angelucci et al., 2015; Damirchi et al., 2018). However, data remain too few, and to date no studies reported IGF-1 or VEGF as significant predictors for CT or CPT improvement.

Thus, the present study which is based on the RCT by Rahe et al. (2015a), but additionally uses 1 year FU data, had two different aims: (i) to compare the effectiveness of CT, CPT, and CPT+C training on cognitive functions 1 year after intervention. The second aim was (ii) to explore predictors of cognitive improvement within the CPT group 1 year after intervention. For this purpose, we analyzed pre-intervention to 1-year FU data of the RCT (Rahe et al., 2015a) in which CT, CPT, or CPT+C were conducted with healthy older adults. Although CPT was not superior to CT concerning short-term effects on cognition (Rahe et al., 2015a), two recent systematic reviews (Zhu et al., 2016; Joubert and Chainay, 2018) came to the conclusion that although data is far from being complete, there is evidence for the superiority of combined cognitive and PT. Therefore, we followed our initial hypotheses: we expected a long-term superiority 1 year after the intervention of CPT (Oswald et al., 2006; Linde and Alfermann, 2014 hypothesis 1). Furthermore, we expected that the effects of CPT with counseling on cognition are superior to that of CPT without counseling in healthy older adults (according to the results of Rahe et al., 2015a hypothesis 2). In an explorative attempt, we also investigated predictors of cognitive improvement within the CPT at FU. Due to the inconsistencies in previous research on predictors of CT improvement and the lack of data on predictors of CPT improvement, no hypotheses were stated for this explorative attempt.

## Materials and Methods

The present study was approved by the ethics committee of the University Hospital Cologne, Germany, and the medical association of Lower Saxony, Germany. It was registered at the WHO ICTRP (ID:DRKS00005194). The study was designed as a multicentre, single-blind RCT. In a former paper, short-term effects of this RCT were reported (Rahe et al., 2015a); methodological details are also described there.

### Participants and Procedure

We recruited healthy older adults in the German cities Cologne, Vechta, and Osnabrück. Individuals were recruited with flyers and posters distributed via health centers, advertisements in the local press, and senior representatives. Participants received no monetary compensation but did not have to pay for the interventions, which are usually fee-based. Individuals interested in the study were first screened for eligibility via phone and then invited for a neuropsychological assessment in which their eligibility was further evaluated. All participants gave written informed consent before the assessment following the Declaration of Helsinki. In case of cardiovascular disease, affirmation for the participation in a physical intervention was obtained from the participants’ general practitioner.

Inclusion criteria were age between 50 and 85 years, normal or corrected-to-normal vision and hearing, and German as the native language. Exclusion criteria were any past or present psychiatric or neurological disease, a condition that prohibited moderate physical activity, past or present intake of psychotropic drugs, and former participation in a cognitive group training. Further exclusion criteria were cognitive impairment as assessed with the cognitive screening DemTect (Kalbe et al., 2004; ≤12 points) and presence of clinically relevant depressive symptoms operationalized with the German version of the Beck Depression Inventory 2 (BDI2; Hautzinger et al., 2009; >19 points). Participants who attended <80% of the training sessions were subsequently excluded from the analyses.

*N* = 81 participants were recruited and allocated to three different training groups at baseline (CT: *n* = 23; CPT: *n* = 28; CPT+C: *n* = 30). The online Research Randomizer^1^ was used to randomize the participants in blocks of three to the intervention types, separately for each study center. Participants were not blinded for group allocation and were told that the study aimed to compare different interventions. The interventions were carried out between October 2012 and June 2013. The single sessions of all nine training groups were led by a trainer (author JM), who was licensed both for the application of CPTs. Cognitive status was assessed at pre-test, post-test, and at FU 1 year after the intervention. For this study only data from pre-test and FU were analyzed.

### Interventions

The three interventions with a maximum of 10 participants per group had a frequency of two sessions per week and a duration of 7 weeks. Each of the 14 sessions lasted 90 min. Training amount was comparable between the three groups. All interventions were described in more detail in a previous report (Rahe et al., 2015a).

#### Group 1: Pure Cognitive Training

Participants of the CT group received the multi-domain CT NEURO*vitalis* (Baller et al., 2009), which was also contained in the other two interventions and which mainly focuses on the age-sensitive domains memory, attention, and executive functions. Every session contains single- and group exercises, activating board games and a short psycho-educational lecture with topics such as “*Relevance of attentional processes*,” “*How does memory work?*,” or “*Planning and problem solving*.” Additionally, participants were asked to perform cognitive homework for ten minutes each day. The cognitive homework of all interventions consisted of a list of 18 different cognitive tasks which could be implemented in the participants’ everyday life (e.g., “Look at a picture of your early childhood and try to collect as many memories related to the picture as possible.” Or “Write down the whole alphabet and try to find a corresponding word that starts with each letter of the alphabet. Use different categories, e.g., animals, vegetables, fruits, professions”). Participants could choose which task they wanted to work on: the only important rule was that they worked on it at least 10 min each day.

The NEURO*vitalis* training has already been shown to be effective for MCI patients (Rahe et al., 2015b), and for patients with Parkinson’s disease (Petrelli et al., 2014, 2015), as well as for patients with dementia (Middelstaedt et al., 2016) in an adopted version for this patient group.

#### Group 2: Combined Cognitive and Physical Training

For CPT interventions, the NEURO*vitalis* training was supplemented with a multi-component physical activity program, which took place in the first 20 min of each session and followed the guidelines for physical interventions by Nelson et al. (2007). The targeted abilities were strength, flexibility, coordination, and endurance. The participants of the CPT had two additional sessions with the psycho-educational contents *physical activity* and *nutrition*. The session *physical activity* covered physical activity recommendations for older adults (adapted from Nelson et al., 2007), which are strategies to increase physical activity in everyday life such as taking the stairs instead of the elevator, and information on positive effects of physical activity on both body and brain health. For practicing at home, the participants received a booklet which illustrated the exercises performed during the training sessions. CPT also targeted nutritional aspects: In the session *nutrition*, the Mediterranean diet (cf. Scarmeas et al., 2006, 2009) was highlighted, and participants received a booklet with information and recipes. To guarantee comparable training duration between the different interventions, the single exercises in the CPT and the CPT+C were given as homework to the participants. Thus, the sessions in each intervention lasted for 90 min.

#### Group 3: Combined Training With Additional Physical Activity Counseling

The CPT intervention with physical activity counseling included additional motivational counseling. Counseling was performed in the first and the last training week according to the approaches of Marcus and Forsyth (2003) and Biddle and Mutrie (2008). It was conducted via two extra single appointments with the trainer in the first and the last training week in which the participants were helped to set goals for their PT and in which they made plans how to achieve them. Based on the results of the fitness test at pre-test, a stage-dependent training schedule with individual exercises, activities, and motivation strategies was generated for each participant. The schedule targeted strength, flexibility, coordination, and endurance. At the end of the program, the trainer and the participant checked which goals were attained and which exercises, activities, or strategies the participant should continue after the training. The trainer used motivational interviewing techniques (Miller and Rollnick, 2004).

### Outcome Measures

Primary cognitive outcomes were assessed with an extensive cognitive test battery in standardized test situations at baseline, post-test, and FU. Assessors had been trained in the test application and scoring and were blinded for training group allocation of the participants.

Primary outcomes of the study were performance changes in the domains of general cognitive status, memory, executive functions, attention, and visuo-construction. Only established test instruments with good test criteria were used. The *general cognitive status* was assessed with the already mentioned DemTect (Kalbe et al., 2004). The immediate and delayed recall of the wordlist subtests of the DemTect (Kalbe et al., 2004) and the delayed recall subtest of the Complex Figure Test (Strauss et al., 2006) were used to measure *verbal memory* and *figural memory*. Within the domain of *executive functions*, *working memory* was assessed with the subtest digit span backward of the German Wechsler Adult Intelligence Scale (Aster et al., 2009), and *verbal fluency* was assessed with the letter verbal fluency tasks S, P, M (total) and the alternating letter verbal fluency task G-R of the Regensburger Wortflüssigkeitstest (RWT, Aschenbrenner et al., 2000) and the semantic verbal fluency tasks supermarket or animal subtest of the DemTect (Kalbe et al., 2004). To assess *planning*, the Zoo Map subtest from the Behavioral Assessment of the Dysexecutive Syndrome (BADS; Wilson, 1996) was used. *Inhibition* was tested using the Stroop test (Bäumler, 1985). For the assessment of *attention*, we used the Brief Test of Attention (BTA, Strauss et al., 2006). The domain *visuo-construction* was assessed using the copy subtest of the Complex Figure Test (Strauss et al., 2006). To minimize retest effects parallel forms of the DemTect (Kalbe et al., 2004) and the RWT (Aschenbrenner et al., 2000) were used, and the use of versions A/B at all assessments (pre-test, post-test, FU) were randomized.

Secondary outcomes were only assessed in the pre- and post-assessment. Therefore, they could not be integrated into the pre-test to FU analyses, but secondary outcomes, assessed at pre-test, could be used as predictors in the regression analyses. These were physical fitness assessed with the Senior Fitness Test (Rikli and Jones, 2001), a reliable and valid measurement of all targeted domains of the conducted physical activity program (strength, endurance, flexibility, and coordination), peripheral blood levels of BDNF, IGF-1, and VEGF, as well as ApoE and BDNF polymorphism. For a detailed overview of the procedure see Rahe et al. (2015a).

### Statistical Analysis

Statistical analyses were performed using IBM SPSS Statistics 25 for Windows (2017). Data were tested for normal distribution with Kolmogorov–Smirnov tests and homogeneity of variances for between-group comparisons with Levene’s tests. For all statistical comparisons, the significance level was set at α = 0.05. The groups were analyzed for differences in the baseline demographic variables age, education, sex, depressive symptoms (BDI 2), apoE4, BDNF, and cognitive status (DemTect) using ANOVAs or Chi-square tests where appropriate. We applied the same procedure to compare participants who could be included in the current analysis and dropouts to FU. Participants who attended less than 80% of the sessions (11 sessions) were not included in the analysis and regarded as drop-outs, to guarantee comparable training duration. *Post hoc* power analyses were performed using G^∗^Power (Faul et al., 2007) to estimate achieved power.

According to the hypothesis and referring to the pre-post comparison of Rahe et al. (2015a), we treated the study as two separate trials comparing CPT vs. CT (hypothesis 1) and CPT vs. CPT+C (hypothesis 2). Change scores for the different outcome measures (general cognitive status, verbal memory, figural memory, working memory, verbal fluency, inhibition, attention, and visuo-construction) from pre-test to FU were analyzed with ANOVAs for repeated measures (rANOVAs), if the assumptions of the ANOVA were fulfilled. The within-subject variable *Time* had two levels (baseline vs. FU). The between-subject variable *Training* also had two levels (trial 1: CPT vs. CT, trial 2: CPT vs. CPT+C). For all cognitive tests, positive change scores indicate better performance at FU, except for the Stroop test, where negative change scores indicate better performances. The effect size partial η^2^ ($\eta_{p}^{2}$) indicates a small ($\eta_{p}^{2}$ > 0.01), moderate ($\eta_{p}^{2}$ > 0.06), or strong effect ($\eta_{p}^{2}$ > 0.14, Field, 2013). Within comparisons were conducted using a *t*-test for pre-test to FU comparisons for each group. If the assumptions of normal distribution and homogeneity of variances were violated or variables were non-parametric, Friedman’s ANOVA was used, and effect sizes ω were reported indicating a small (ω > 0.10), moderate (ω > 0.30), or strong effect (ω > 0.50; Field, 2013).

We calculated predictions of cognitive improvement only for CPT to compare the data to those presented for the pre- post-test results by Rahe et al. (2015a). Predictors of cognitive improvement were calculated using backward multiple regressions. The change scores (FU – pre-test) of cognitive variables were calculated. According to the current literature and following the analysis conducted by Rahe et al. (2015a), the predictors age, education, sex, baseline cognitive scores, ApoE, and BDNF polymorphisms, baseline levels of BDNF, IGF-1, and VEGF, and baseline overall fitness were integrated into the regression analyses. The assumptions of multiple regression models were checked according to the suggestions of Field (2013).

## Results

### Participants’ Flow and Characteristics

**FIGURE 1:**
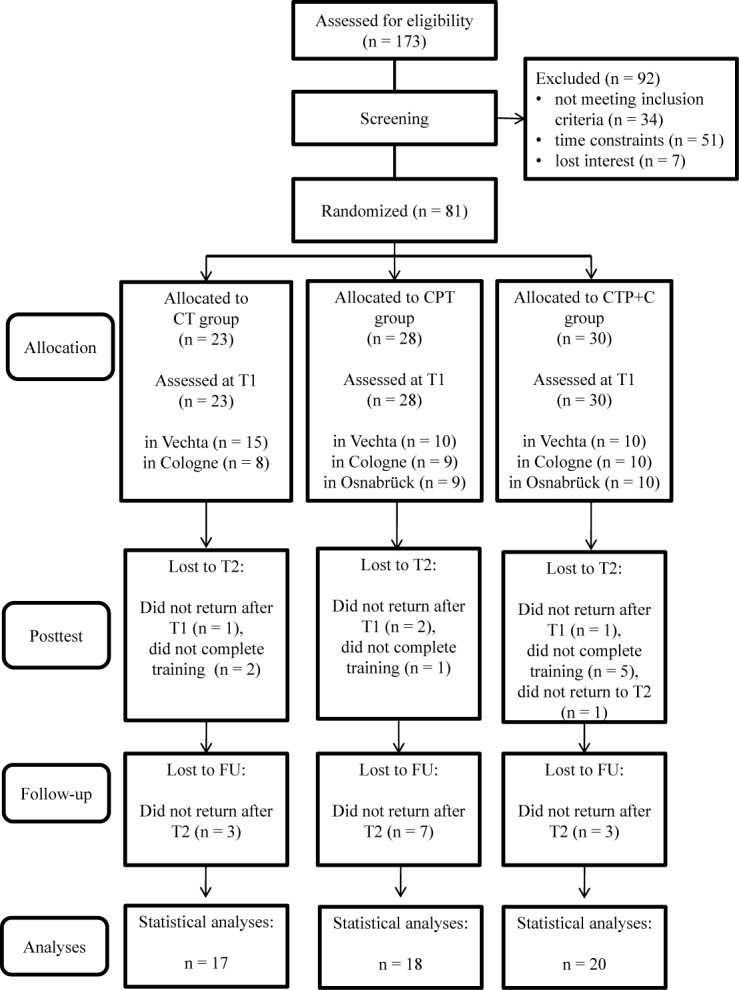
Participants’ Flow throughout the Study.

In sum, *N* = 81 participants fulfilled inclusion criteria at pre-test, were randomized to one of the three interventions, and attended at least one training session. As participants for only two training groups could be recruited in Osnabrück, an additional group was trained in Vechta. A total of *n* = 26 participants dropped out until FU because of health issues, personal reasons, time constraints or less than 80% attendance of sessions. Figure 1 shows the participants’ flow through the study. For the statistical analysis, the data of the remaining *N* = 55 participants were used. Participants of the three intervention groups did not differ significantly in the baseline demographic variables age, education, and sex, nor in the apoE and BDNF genotype, nor the handedness, nor in the FU variables age and cognitive status (all *p* > 0.05, see Table 1). Also, participants and drop-outs of the study did not differ significantly in the pre-test demographics (all *p*> 0.05, see Table 2), except in the CT group, where participants who dropped out of the study showed a significantly lower score in the DemTect total score at pre-test, *t*(21) = 3.47, *p* = 0.002.

**Table 1:** Baseline characteristics of the study sample, which was assessed at FU1.

Demographics	CT (*n* = 17)				CPT (*n* = 18)				CPT+C (*n* = 20)				
	*M*	(*SD*)		Range	*M*	(*SD*)		Range	*M*	(*SD*)		Range	*p*
Age at Baseline	67.53	(5.88)		53–74	68.22	(7.96)		51–81	68.75	(6.62)		50–79	0.866^a^
Age at FU	68.35	(6.01)		53–76	69.00	(7.79)		51–81	69.40	(6.57)		51–81	0.879^a^
Education	14.53	(2.90)		11–20	14.44	(3.45)		11–22	14.60	(3.68)		11–21	0.990^a^
CS at Baseline	16.76	(1.56)		13–18	16.22	(1.73)		13–18	16.80	(1.77)		13–18	0.518^a^
CS at FU	16.76	(1.56)		13–18	16.22	(1.73)		13–18	16.80	(1.77)		13–18	0.518^a^
Depression Baseline	5.47	(3.89)		1–12	6.61	(4.08)		0–16	6.15	(4.64)		1–16	0.728^a^
Depression at FU	6.06	(4.84)		0–13	6.06	(4.56)		0–17	6.20	(5.98)		0–23	0.995^a^
Handedness	right: 16	left: 0		mixed: 1	right: 17	left:0		mixed: 1	right: 18	left: 0		mixed: 2	0.840^b^
apoE genotype	E4-Carrier: 2				E4-Carrier: 6				E4-Carrier: 5				0.319^b^
BDNF genotype	Val66Met: 6				Val66Met: 9				Val66Met: 10				0.599^b^
Sex	** 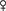 ** = 10 ** 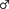 ** = 7				** 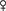 ** = 11 ** 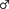 ** = 7				** 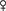 ** = 15 ** 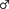 ** = 5				0.526^b^
	58.8% 41.2%				61.1% 38.9%				75.0% 25.0%				

*Range of DemTect norms for normal cognitive status: 13–18. apoE, Apolipoprotein E; BDNF, brain-derived neurotropic factor; CT, Cognitive training; CS, Cognitive Status; CPT, Cognitive training with additional physical activity; CPT+C, Cognitive training with additional physical activity and counseling. ^*a*^Comparison of groups at baseline with ANOVAs. ^*b*^Comparison of groups at baseline with Chi-square tests*.

**Table 2:** Comparison of dropouts (from baseline to FU) and participants at baseline characteristics.

Demographics	CT (*n* = 23)			CPT (*n* = 28)			CPT+C (*n* = 30)		
	Dropouts (*n* = 6)	Participants (*n* = 17)	*p*	Dropouts (*n* = 10)	Participants (*n* = 18)	*p*	Dropouts (*n* = 10)	Participants (*n* = 20)	*p*
Age	67.67 (10.05)	67.53 (5.89)	0.968^a^	68.90 (5.47)	68.22 (7.96)	0.831^a^	67.50 (9.61)	68.75 (6.62)	0.678^a^
Education	15.17 (2.48)	14.53 (2.90)	0.637^a^	14.50 (3.24)	14.44 (3.45)	0.967^a^	13.40 (2.88)	14.60 (3.68)	0.375^a^
CS	13.50 (2.95)	16.76 (1.56)	**0.002^a^**	16.70 (2.16)	16.22 (1.73)	0.528^a^	16.80 (2.01)	16.80 (1.77)	1.000^a^
apoE genotype	E4-Carrier: 0	E4-Carrier: 2	0.379^b^	E4-Carrier: 3	E4-Carrier: 6	0.873^b^	E4-Carrier: 0	E4-Carrier:5	0.261^b^
BDNF genotype	Val66Met:2	Val66Met: 6	0.931^b^	Val66Met: 4	Val66Met: 9	1.00^b^	Val66Met: 0	Val66Met: 10	0.064^b^
Sex	** 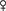 ** = 4 ** 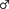 ** = 2	** 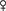 ** = 10 ** 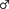 ** = 7	0.735^b^	** 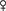 ** = 8 ** 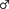 ** = 2	** 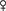 ** = 11 ** 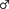 ** = 7	0.305^b^	** 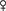 ** = 7 ** 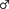 ** = 3	** 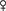 ** = 15 ** 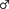 ** = 5	0.770^b^
	66.7%; 33.3%	58.8%; 41.2%		80.0%; 20.0%	61.1%; 38.9%		70.0%; 30.0%	75.0%; 25.0%	

*Range of DemTect norms for normal cognitive status: 13–18. apoE, Apolipoprotein E; BDNF, brain-derived neurotropic factor; CT, Cognitive training; CS, Cognitive Status; CPT, Cognitive training with additional physical activity; CPT+C, Cognitive training with additional physical activity and counseling. ^*a*^Comparison of groups at baseline with *t*-tests. ^*b*^Comparison of groups at baseline with Chi-square tests. Significant *p*-values are displayed in bold letters*.

### Statistical Power of Group Differences in Outcome Measures

Baseline to FU performances of the intervention groups are shown in Table 3. We achieved a 21% power to detect small interaction effects ($\eta_{p}^{2}$ > 0.01), 82% power to detect moderate interaction effects ($\eta_{p}^{2}$ > 0.06), and 99% power to detect strong interaction effects ($\eta_{p}^{2}$ > 0.14) (Cohen, 1988), in the comparison of the groups for testing hypothesis 1 (*N* = 35, 2-tailed α = 0.05). For testing hypothesis 2 (CPT vs. CPT + C), a 22% power to detect small interaction effects ($\eta_{p}^{2}$ > 0.01), 85% power to detect moderate interaction effects ($\eta_{p}^{2}$ > 0.06), and 99% power to detect strong interaction effects ($\eta_{p}^{2}$ > 0.14) was achieved (*N* = 38, 2-tailed α = 0.05).

**Table 3:** Primary outcomes of the training groups at pre-test and FU.

Domain		CT (*n* = 17)		H1	CPT (*n* = 18)		H2	CPT+C (*n* = 20)	
		*M* (*SD*)			*M* (*SD*)			*M* (*SD*)	
	Maximum	Pre-test	FU^b^	*p^c^*	Pre-test	FU^b^	*p^d^*	Pre-test	FU^b^
**Memory**					
**Verbal Memory**					
DemTect, *IR*	20	13.88 (2.37)	15.12 (2.28)		13.78 (2.26)	15.44** (2.33)	^∗^	13.95 (2.56)	14.40 (2.19)
DemTect, *DR*	10	6.06 (2.33)	5.65 (2.61)		5.22 (2.29)	6.17 (2.46)		6.50 (2.31)	5.90 (2.65)
**Figural Memory**					
CFT, *DR*	1	21.00 (6.22)	24.24** (5.01)		20.17 (6.47)	24.22*** (5.20)		19.50 (5.37)	22.82** (6.52)
**Attention**					
BTA	20	17.41 (2.79)	18.53* (1.46)		15.89 (2.40)	17.94*** (1.89)		17.25 (2.81)	18.35* (1.66)
**Executive Functions**					
**Working memory**					
WAIS-II, *DSB*	14	6.88 (1.50)	7.82 (2.27)		6.78 (2.69)	8.06 (2.58)		6.80 (1.67)	7.60 (2.28)
**Verbal fluency**					
RWT, *total*	–	44.88 (12.02)	41.65 (9.52)		43.11 (11.35)	42.94 (10.69)		45.20 (11.55)	48.00 (12.00)
RWT, *G-R*%	90%	51.59 (31.56)	51.65 (27.91)		62.72 (29.87)	63.78 (28.54)		53.05 (32.69)	75.63** (21.74)
**Inhibition**					
Stroop Diff.^a^	–	48.71 (17.40)	45.17 (15.51)		40.25 (19.82)	39.73 (15.61)		45.63 (15.55)	42.30 (10.87)
**Planning**					
Key Search	16	13.76 (2.22)	13.29 (2.62)		11.78 (3.47)	12.22 (2.86)		12.55 (2.72)	13.15 (2.50)
**Cognitive Status**					
DemTect	18	16.76 (1.56)	16.65 (2.03)		16.22 (1.73)	17.06 (1.39)	^∗^	16.80 (1.77)	16.60 (1.93)
**Visuo-construction**					
CFT, *Copy*	36	34.24 (1.39)	34.59 (2.37)		34.61 (1.58)	35.22 (1.52)		33.60 (3.00)	34.00 (4.89)

*The DemTect is from Kalbe et al. (2004). The Complex Figure Test (CFT) and the Brief Test of Attention (BTA) as described in Strauss et al. (2006) were used. The German Wechsler Adult Intelligence Scale (WAIS-II) is from Aster et al. (2009). The trials *S*, *P*, *M* (total) and alternating *G-R* of the Regensburger Wort Flüssigkeits-Test (RWT) from Aschenbrenner et al. (2000) were used. The Stroop Test is from Bäumler (1985). The Key Search is from Wilson (1996). FU, Follow-up; CT, Cognitive training; CPT, Cognitive training with additional physical activity; CPT+C, Cognitive training with additional physical activity and counseling; DR, subtest *delayed recall*. DSB, subtest *digit span backward*; *G-R*%, percentile rank for subtest *G-R*; IR, subtest *immediate recall*; H1, hypothesis 1 (CPT vs. CT); H2, hypothesis 2 (CPT vs. CPT+C); NT, subtest *number transcoding*; S/A, subtest *supermarket / animal*. Stroop Diff. = [trial 3 -*M* (trial 1 + trial. 2)] (see van Hooren et al., 2007). ^*a*^Smaller scores indicate better performance. ^*b*^*p*-values of with-in comparisons for each group. ^*c*^*p*-values of comparison for hypothesis 1 (H1) *Time* × *Training* interaction (CPT vs. CT). ^*d*^*p*-values of comparison for hypothesis 2 (H2) *Time* × *Training* interaction (CPT vs. CPT+C). ^∗^*p* ≤ 0.05; ^∗∗^*p* ≤ 0.01; and ^∗∗∗^*p* ≤ 0.00*.

### Cognitive Outcomes

rANOVAs were used to analyze the differences between groups in all primary outcomes. As indicated in the methods section, the within-subject variable *Time* had two levels, and the between-subject variable *Training* also had two levels (trial 1: CPT vs. CT, trial 2: CPT vs. CPT+C). A significance of the within-subject variable *Time* indicates cognitive changes for each group.

#### Hypothesis 1: CT vs. CPT

No significant *Time × Training* interactions were found when comparing CPT vs. CT. Overall analyses revealed significant within-subject effects of *Time* for verbal short-term memory, [*F*(1,33) = 12.47, MSE = 36.81, *p* = 0.001, $\eta_{p}^{2}$ = 0.27], figural memory [*F*(1,33) = 25.73, MSE = 232.37, *p* < 0.001, $\eta_{p}^{2}$ = 0.44], working memory [*F*(1,33) = 15.35, MSE = 21.52, *p* < 0.001, $\eta_{p}^{2}$ = 0.32], and attention [*F*(1,33) = 20.65, MSE = 44.02, *p* < 0.001, $\eta_{p}^{2}$ = 0.39], indicating better performance at FU. No significant between-subject effects of the factor *Training* were found.

#### Hypothesis 2: CPT vs. CPT+C

**FIGURE 2:**
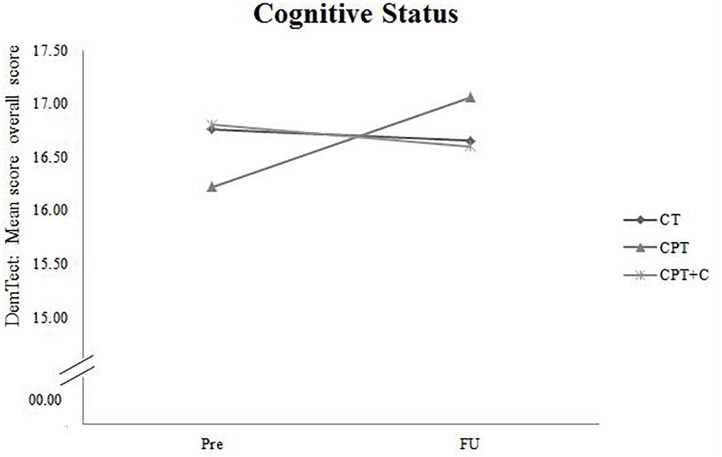
Pre- and FU results of the domain “Cognitive Status” measured with the overall score of the DemTect for each of the three interventions.

**FIGURE 3:**
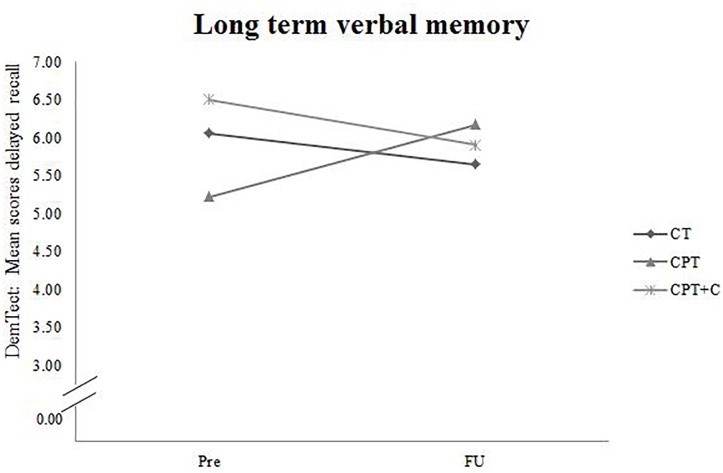
Pre- and FU results of the domain “Long term verbal memory” measured with the subtest for delayed recall of the DemTect for each of the three interventions.

A significant *Time × Training* interaction was found when comparing CPT vs. CPT+C for the general cognitive status, *F*(1,36) = 4.94, MSE = 5.08, *p* = 0.033, $\eta_{p}^{2}$ = 0.12, and verbal long-term memory, *F*(1,36) = 6.91, *MSE* = 11.30, *p* = 0.013, $\eta_{p}^{2}$ = 0.16 in favor of the CPT group. These results are illustrated in Figures 2, 3. However, also a trend (*p* < 0.01) was shown for alternating verbal fluency, measured with the RWT G-R test [*F*(1, 36) = 3.94, MSE = 2140.99, *p* = 0.055, $\eta_{p}^{2}$ = 0.10], favoring the CPT+C training. Overall analyses revealed significant within-subject effects of Time for verbal short-term memory, *F*(1,36) = 7.84, MSE = 21.22, *p* = 0.008, $\eta_{p}^{2}$ = 0.18, figural memory, *F*(1,36) = 30.21, MSE = 258.03, *p* < 0.001, $\eta_{p}^{2}$ = 0.46, working memory, *F*(1,36) = 12.19, MSE = 20.45, *p* = 0.001, $\eta_{p}^{2}$ = 0.25, verbal fluency, *F*(1,36) = 4.75, MSE = 2581.59, *p* = 0.036, $\eta_{p}^{2}$ = 0.12, and attention, *F*(1,36) = 21.13, MSE = 47.17, *p* < 0.001, $\eta_{p}^{2}$ = 0.37. No significant between-subject effects of the factor *Training* were found.

#### Within Comparisons for Training Groups

*T*-tests were calculated to further investigate within comparisons for each of the three training groups. Alpha-correction was performed within each domain.

In the CT group there were significant within effects in the domains figural memory [*t*(16) = -2.92; *p* = 0.010], showing an improvement from baseline (*M* = 21.00; *SD* = 6.22) to FU (*M* = 24.24; *SD* = 6.47), and attention [*t*(16) = -2.16; *p* = 0.045], also showing that participants improved from baseline (*M* = 17.41; *SD* = 2.79) to FU (*M* = 18.53; *SD* = 1.46). These improvements go beyond the expected retest effects, which are 0.89 points for the Complex figure rest (figural memory, Strauss et al., 2006), and 0.70 for the BTA (attention, Strauss et al., 2006).

In the CPT group, there were significant within-effects for the domain verbal short term-memory [*t*(17) = -3.30; *p* = 0.004, baseline: *M* = 13.78; *SD* = 2.26; FU : *M* = 15.44; *SD* = 2.33], figural memory [*t*(17) = -4.39; *p* < 0.001, baseline: *M* = 20.17; *SD* = 5.01; FU : *M* = 24.22; *SD* = 5.20], and attention [*t*(17) = -4.33; *p* < 0.001, baseline: *M* = 15.89; *SD* = 2.40; FU : *M* = 17.94; *SD* = 1.89]. All these domains show an improvement of the performance from baseline to FU which again go beyond the expected retest effects.

Results of the within comparisons in the CPT+C group also show significant effects for the domains figural memory [*t*(19) = -3.44; *p* = 0.003, baseline: *M* = 19.50; *SD* = 5.37; FU : *M* = 22.82; *SD* = 6.52], as well as for letter verbal fluency [*t*(18) = -3.00; *p* = 0.008, baseline: *M* = 53.05; *SD* = 32.69; FU : *M* = 75.63; *SD* = 21.74], and attention [*t*(19) = -2.24; *p* = .037, baseline: *M* = 17.25; *SD* = 2.81; FU : *M* = 18.35; *SD* = 1.66]. Again, all these three domains show an improvement from baseline to FU with improvements larger than the expected retest effects for all domains – which are between 0.76 and 0.82 for letter verbal fluency measured with the raw scores of the RWT (Aschenbrenner et al., 2000).

### Predictors of Cognitive Improvement Within CPT

As indicated in the Methods section, we used backward multiple regressions to analyze predictors of cognitive improvement within the CPT group. Thereby, the achievement of the best model fit can be ensured while taking into account every relevant predictor. This study had a 35% power to detect a large effect (*f^2^* = 0.35, assuming a maximum of four predictors, based on n = 18 participants at the CPT group at FU, Field, 2009). Therefore, correlations between all ten predictor variables and (...) the different outcomes (difference scores of: general cognitive state (....), verbal short- and long-term memory, figural memory, working memory, verbal fluency, alternating letter verbal fluency, inhibition, attention, planning, and visuo-construction) were calculated. Variables that had a significant correlation with the outcome variable were integrated into the regression model assuming a maximum of four predictors. In cases, where more than four variables had a significant correlation with the outcome variable, the four variables with the highest significant correlation with the outcome variable were used.

The primary results of the predictor analyses within the CPT group are: (i) lower baseline performance was a predictor of gains in verbal short-term memory (ß = -0.44), letter verbal fluency (ß = -0.59), alternating verbal letter fluency (ß = -0.40), and attention (ß = -0.64), (ii) a lower educational level was a predictor of gains in working memory (ß = -0.59), (iii) low blood levels of BDNF were predictive of an improvement in alternating letter verbal fluency (ß = -0.35), and (iv) higher blood levels of IGF-1 were also predictive of an improvement in alternating letter verbal fluency (ß = 0.36). Statistical details are shown in Supplementary Table 1.

## Discussion

The main findings of our study are that (i) no significant interaction effect which supports our hypothesis 1 – that CPT is superior to CT – could be found, but it should be noted that cognitive gains could be observed in both groups in verbal short-term memory, figural memory, working memory, and attention. Furthermore, (ii) referring to our hypotheses 2 – that effects of CPT with counseling on various cognitive domains are superior to that of CPT without counseling in healthy older adults -, there was only a trend for significance for a *Time* × *Training* effect in favor of the CPT+C group for alternating verbal fluency, while significant interaction effects *Time*× *Training* in favor of the CPT group compared to the CPT+C group were found for the general cognitive status and verbal long-term memory. Finally, our results show that (iii) low cognitive baseline performance, low education, low blood baseline levels of BDNF, and high blood baseline levels of IGF-1, at least in part, predict an improvement of cognitive functions 1 year after a CPT intervention.

Yet, this is one of the first studies comparing the effectiveness of CT, CPT, and CPT+C training on cognitive functions 1 year after intervention, as long-term effects are the main goal for interventions on cognition in healthy older adults (Zhu et al., 2016) and have rarely been studied. The previous study by Rahe et al. (2015a) only compared pre-test and post-test data.

### Discussion of Hypothesis 1: CT vs. CPT

No significant interaction effects favoring CPT in comparison to pure CT could be observed. This fact contradicts our hypothesis 1 that CPT is superior to CT, but is in line with the findings of the pre-post comparison (Rahe et al., 2015a) and other RCTs failing to support the superiority of CPT at pre-post comparison (Legault et al., 2011; Barnes et al., 2013; Shatil, 2013) and pre-FU comparisons (Linde and Alfermann, 2014). In the meta-analysis of Zhu et al. (2016), a preliminary analysis of three studies (due to limited data) also showed no significant effects for the comparison between CPT and CT. Oswald et al. (2006), demonstrated that CT and CPT can improve cognitive performance in short-term as well as in the long-term in the outcome measure attention. The intervention groups in that study were contrasted with a passive control group, while the direct contrast (CPT vs. CT) was not analyzed. Our results are further supported by an exergaming study of Anderson-Hanley et al. (2018), who also found that physical activity with high and with low cognitive challenge yielded significant moderate effects on executive function, yet there was no significant interaction. In summary, the question of whether or not CPT is favorable concerning cognitive outcome cannot be answered conclusively, but our data support the notion that both trainings yield comparable results.

Besides the question which training is superior, it should be noted that both trainings, CPT and CT, can be regarded as efficient in stabilizing or even enhancing cognitive functions in healthy older adults at post-test (Kelly et al., 2014a; Law et al., 2014). Our data provide evidence that this is still the case 1 year after intervention, indicated by the found *Time* effects in both groups. Also, our conducted with-in comparison showed cognitive improvements for both types of training (figural memory and attention in both types of training, and additionally, verbal short-term memory after CPT) that go beyond expected retest effects. Also, the ACTIVE trial, the study with the largest sample of healthy older adults participating in CT, showed improvements in targeted cognitive abilities after reasoning and speed training (but not memory training) over a very long period of 10 years after the intervention (Rebok et al., 2014). However, as maintenance or even improvement of cognitive functions is a crucial indicator for intervention efficiency (Zhu et al., 2016), more studies with FU are needed to confirm the long-term effects of combined interventions and CT (e.g., Fabre et al., 2002; Legault et al., 2011; Theill et al., 2013).

### Discussion of Hypothesis 2: CPT vs. CPT+C

A significant *Time*× *Training* interaction was found when comparing CPT vs. CPT+C in the domains of general cognitive status and verbal long-term memory in favor of the CPT group. This finding is not in line with the results of the pre-post comparison of the data, in which Rahe et al. (2015b) did not find significant interaction effects in favor of the CPT. However, that study showed a significant effect in alternating letter verbal fluency and planning, favoring the CPT+C group, and we also found a trend (*p* < 0.01) for the alternating verbal fluency, measured with the RWT G-R test, favoring the CPT+C training group in the FU analysis. We can only speculate about the reasons for this pattern. As discussed by Rahe et al. (2015b) it is possible that the CPT+C group was involved on a more “cognitive level” than the CPT group, because their task was to plan physical activity strategically, in contrast to the CPT group, which focused particularly on the behavioral level of being physically active. This hypothesis could explain the trend observed in the alternating letter verbal fluency task, favoring the CPT+C even 1 year after the intervention, as this task requires the use of a strategy (Hughes and Bryan, 2002). According to Rahe et al. (2015a) it is possible that participants’ complaints about higher strains caused by the additional individual counseling sessions in the CPT+C training might have resulted in less training motivation, therefore in less training and consequently fewer benefits compared to the CPT. Accordingly, it may be possible that a concrete training plan without additional individual effort in planning is more appropriate and more efficient for participants who are not highly motivated to individualize their training. In contrast, as “personal training” is an emergent training concept, it can be presumed that there are individuals for who CPT+C will be suitable and even more efficient than CPT. Future research is needed to elaborate on this topic. With regard to the interaction effect in favor of the CPT for the general cognitive status and verbal long-term memory, which is reflected in improved scores at FU measurements for the CPT group on a descriptive level, while the CPT+C group did not improve (and even slightly declined in verbal long-term memory), these data emphasize the potential of a CPT to enhance cognition in long-term. However, as for the comparison of CT versus CPT, it should be noted that within-comparisons indicated cognitive improvements for both types of training - figural memory and attention for both CT and CPT, short term memory for CPT, and verbal fluency for the CPT+C.

### Discussion of Predictor Analysis Within CPT

This is the first study that investigated predictors within a CPT 1 year after intervention. In line with the results of the pre-post prediction (Rahe et al., 2015a), a lower initial baseline performance predicted gains in the domains verbal short-term memory, letter verbal fluency, alternating letter verbal fluency, and attention. This finding is also consistent with several studies showing that lower initial baseline performance is predictive of CT improvement in healthy older adults (e.g., Whitlock et al., 2012; Rahe et al., 2015a). However, also contradictory results exist (e.g., Fairchild et al., 2013), showing that participants with an initially higher baseline benefit most from CT. It is important to consider the different statistical analyses used and to differentiate between the prediction of short and long-term effects when interpreting the different patterns of results (e.g., use of backward or forward multiple regression, latent growth models, etc.,) which has not been systematically conducted yet.

The fact that an initial low baseline performance was predictive in the present study might be explained by the compensation hypothesis (Lövdén et al., 2012). The compensation account implies that participants, who are already functioning at optimal levels, have less room for improvement in CT performance. Accordingly, participants who start with an initially lower performance will gain more from CT. It is not entirely clear under which conditions this account may or may not be applicable (Lövdén et al., 2012). Lövdén et al. (2012) showed that one factor that permits predictions about the empirical condition under which compensation is more likely to occur may be the theoretical distinction between flexibility [which “denotes the capacity to optimize performance within the limits of the brain’s currently imposed structural constraints”; Lövdén et al., 2010) and plasticity (the capacity for changes in the possible range of cognitive performance enabled by flexibility (e.g., Baltes, 1987; Baltes et al., 2006)]. Applying this theory to our results, one could argue that if the brain’s performance for a task was already optimized within current structural constraints, then fewer benefits could be expected from the CT and CPT. Therefore, participants with an initially high baseline performance did not profit directly right after the training, while participants with an initially low baseline performance did so, because the extensive practice pushed these participants beyond their initial range of performance, thereby inducing plastic changes (Lövdén et al., 2012). Another reason that may explain the results can be that the training was too easy for the participants with an initially high baseline performance, resulting in less training gain based on a lack of challenge.

In the present study, lower education was a significant predictor for a gain in the domain of working memory. There are only a few studies, which investigated the association between education and memory training gains, but yet, most of them did not find significant results (e.g., Dorfman and Ager, 1989; Wolters et al., 1996). However, Rebok et al. (2013) found a significant association, indicating that healthy older adults with more education show improvement in memory performance, a finding that is at odds with our data. Notably, one can conceive of explanations for both directions of the effect. On the one hand, it could be possible that individuals with more education benefit most from CT and CPT, as education is a driver of cognitive and neural plasticity (Mandolesi et al., 2017). On the other hand, it could also be possible, according to the compensation hypothesis (Lövdén et al., 2012), that healthy older adults with less education may benefit more, as there is more “room for improvement” (see above). Furthermore, in some tests ceiling effects are possible, so that for participants who show ceiling effects, there is no room for improvement (our data indicate that this could be the case, for example, for the visuo-constructive task, i.e., copying the Rey Complex Figure). Longitudinal studies are needed to determine whether education affects the maintenance of CPT gains and in which way CT gains are affected (Langbaum et al., 2009).

Interestingly, as already shown in the study of Rahe et al. (2015a), lower initial blood levels of BDNF were a predictor of an improvement in the domain alternating letter verbal fluency, showing that for this executive task BDNF level was essential for training-induced plasticity. However, contrary results exist (Voss et al., 2013), showing that higher blood levels of BDNF were associated with a greater change in functional connectivity as measured with structural and functional MRI in a group which was trained with non-aerobic exercises. In that study, however, the relationship between growth factors and cognitive outcomes was not assessed and, therefore, the association of changes of growth factors with cognitive outcomes still needs more investigation. BDNF is highly concentrated in the hippocampus, but is also distributed throughout the entire brain (Murer et al., 1999). BDNF is considered to mediate the effects of exercise on synaptogenesis, synaptic plasticity, and enhanced learning and memory (Cotman et al., 2007). Therefore, the expression of BDNF plays an essential role in regions that are vital to learning and memory (Bekinschtein et al., 2008). In particular, BDNF is critical for synaptic plasticity and memory-processing in the adult brain (Alonso et al., 2002; Tyler et al., 2002), because it induces long-term potentiation in the hippocampus, which is a form of synaptic plasticity thought to underlie long-term potentiation formation (Izquierdo and Medina, 1997). A recent systematic review on the effect of BDNF polymorphisms on cognition could show that several studies report an association between the Val66Met polymorphism (which is one of the most extensively studied single-nucleotide polymorphism in the BDNF gene, see Chen et al., 2006) and changes of various cognitive domains (Toh et al., 2018). More precisely, the highest percentage of positive associations were shown between Val66Met polymorphism and memory (41.3%), followed by executive functions (38.3%), and attention and concentration (17.4%). However, the neurobiological mechanisms for these benefits are not fully understood, and the reason why we found that BDNF was only a predictor of executive functions remains to be further investigated. Rahe et al. (2015a) could also show a moderation effect indicating that *change in physical fitness × change in BDNF* significantly predicted gains in this task suggestive of high changes in physical fitness being predictive of high cognitive gains and a strengthening of this association due to higher BDNF changes. However, we could not test this assumption for FU data, because data on BDNF and physical fitness at FU were not assessed.

Higher initial blood levels of IGF-1 predicted improvement in the alternating letter verbal fluency task in FU assessment. This result can be interpreted in line with previous studies reporting the association between IGF-1 and functional connectivity change (Voss et al., 2013). The IGF-1 plays an important role in the regulation of adult neurogenesis (e.g., Fernandez and Torres-Alemán, 2012; O’Kusky and Ye, 2012; Ziegler et al., 2015). A study of Liu et al. (2009) showed that the conditional deletion of the IGF-receptor gene in mice results in an almost complete loss of the dentate gyrus, which is part of the hippocampus and essential for learning and memory. Higher levels of IGF-1 seem to play an essential role in benefits from CT, although we found an effect on executive functions, rather than memory. Since the present and the original study of Rahe et al. (2015a) are the first that investigated the association between effects of a combined lifestyle interventions on growth factors, our results warrant further investigation.

In the present study, apoE4 was not predictive for any of the investigated outcomes, even though in the pre- post-test comparison of Rahe et al. (2015a), the apoE polymorphism was predictive for performance in an alternating letter verbal fluency task, indicating that non-carriers of the apoE 4 allele showed gains in executive functions compared to carriers of the ApoE 4 allele. Yet, there was an ever smaller sample in the FU sample for apoE4 carriers than in the pre-test – post-test comparison, which may explain the missing effect; further studies with larger sample sizes will have to shed light on the potential effect of apoE4 on training effects in healthy older adults.

Overall, the identification of predictors of cognitive interventions gains increasing interest to elucidate the question of who will benefit from CT or CPT interventions to optimize interventions for specific target groups in the future.

### Limitations

Some limitations have to be considered when interpreting our data. One limitation is the relatively small sample size in general and due to dropouts to FU: power analysis indicated a power of only 21% (hypothesis 1) and 22% (hypothesis 2) to detect small interaction effects, so that studies with larger sample sizes might be able to detect more differences between the effects of the different types of interventions.

Furthermore, our sample was highly educated, and our sample also represents highly motivated and active healthy elderly. As outlined by Rahe et al. (2015a), this constitutes a problem affecting most intervention studies of healthy older people, as participating in a study is always voluntary and it can be assumed that (...) volunteers differ from non-volunteers, not only in (...) motivation but also in outcome expectation, socio-demographic variables, and healthy lifestyles (Oswald et al., 2006; Unverzagt et al., 2009; Schubert et al., 2014). Also, our eligibility criteria might have limited the variability in our sample, e.g., no homozygote E4/E4-carrier was identified. This could be since homozygote E4/E4 carrier, who have the highest risk to develop cognitive dysfunctions or dementia, may have been excluded as a result of our screening with the DemTect (Kalbe et al., 2004). Notably, in the CT group, six individuals dropped out before FU, who had a significantly lower DemTect score at baseline (Table 2). Reasons for their refusal to participate in the FU can only be speculated, but it is possible that these individuals had developed cognitive decline, and might have been demotivated to participate in the FU. However, this aspect needs further research.

An overview about other activities in which the participants of all three groups might have engaged during the study period and the time period until the FU test was not obtained, so that we are unable to analyze which training (especially in the comparison of CPT and CPT+C) ultimately led to more physical activities in the long term. Detailed protocols of further off-study activities, as performed by, e.g., Graessel et al. (2011) in patients with dementia, which help to estimate their possible influence and protocols assessing leisure physical activity (e.g., self-report questionnaires or a physical activity diary) should be integrated into future studies. Besides, our study did not measure physical activities and peripheral growth factors at FU. Therefore, we could not conduct FU analyses for the secondary outcome parameters physical fitness and peripheral growth factors, as in the original study (Rahe et al., 2015a).

A further limitation of the study was the fact that we did not check how long and to what extend the participants conducted their homework. For future studies, a homework-diary should be implemented to control for intensity and total time spent on the cognitive homework as a further possible factor influencing CT performance.

A passive control group was not included in the original study so that cognitive improvements induced by any of our training types can only be estimated in a limited way. However, the focus of the study was on the hypothesis that the combination of CT with other lifestyle factors is superior to pure CT and to compare the results of the pre-post comparison to the pre-FU comparisons of the conducted training.

As a final limitation, there were some constraints in the blood sampling procedure (analyses were conducted in three different laboratories, no standardization of time of blood withdrawal) and the pre-analytics for blood analyses, which are described in more detail in Rahe et al. (2015a).

## Conclusion

Summarized, our 1 year FU data did not show additional effects of CPT compared to pure CT on cognitive functions in healthy older adults so that no clear recommendation can be given with regard to the (...) type of training and cognitive outcome (although CPT surely has the advantage to include physical activity). CPT is probably more suitable for the majority of healthy older people compared to CPT+C, as it showed more long-term improvement in the general cognitive status and verbal long-term memory – two domains which are highly relevant in the context of aging and cognition. Importantly, it should be noted that within-group comparisons indicate that any training is helpful to maintain cognition.

The finding that participants with lower initial baseline scores in the tested domains benefitted more from the CPT in our study indicates that this might be an important target group for such interventions. Likewise, CPT might be especially productive for individuals with low education. The fact that peripheral growth factors also predict CT outcome points to the mechanisms underlying cognitive plasticity – a topic which should be pursued further.

A particular strength of this investigation is the fact that the present study is one of the first that investigates the long-term effects of CT vs. CPT training and therefore adds to the existing literature. The results of future RCTs with long-term data assessment on single and combined CT will have to shed further light on the underlying mechanisms of non-pharmacological interventions to stabilize cognition.

## Data Availability Statement

Datasets are available on request. The raw data supporting the conclusions of this manuscript will be made available by the authors, without undue reservation, to any qualified researcher.

## Author Contributions

JM, EK, and JK were responsible for the recruitment of patients included in the study. All authors were responsible for the conceptualization and design of the project, and contributed to the interpretation of the data presented in the articles and revision of the manuscript. EK and JK were responsible for the supervision and coordination. MR was responsible for the statistical analyses. MR, EK, and JM wrote the final version of the article.

## Conflict of Interest Statement

EK and JK are authors of the NEUROvitalis Program. The remaining authors declare that the research was conducted in the absence of any commercial or financial relationships that could be construed as a potential conflict of interest.

## Footnotes

1 http://www.randomizer.org
