# Supplementary material for: Effects of a Cognitive Training With and Without Additional Physical Activity in Healthy Older Adults: A Follow-Up 1 Year After a Randomized Controlled Trial
Source: Front Aging Neurosci. 2018 Dec 18;10:407. doi: 10.3389/fnagi.2018.00407 (PMC6305338; doi:10.3389/fnagi.2018.00407)
Supplement: Supplementary file 1 [file Table_1.docx]

Supplementary Material

Effects of a Cognitive training with and without additional physical activity in healthy older adults: a follow-up one year after a randomized controlled trial

Elke Kalbe^a1^, Mandy Roheger^a1^, Kay Paluszak^a^, Julia Meyer^b^, Jutta Becker^c^, Gereon R. Fink^d^, Juraj Kukolja^e^, Andreas Rahn^f^, Florian Szabados^g^, Brunhilde Wirth^c^, & Josef Kessler^h^

^a^ Department of Medical Psychology | Neuropsychology and Gender studies & Center for Neuropsychological Diagnostics and Intervention (CeNDI), University Hospital Cologne, Kerpener Str. 68, 50937 Cologne, Germany; elke.kalbe@uk-koeln.de; mandy.roheger@uk-koeln.de; +49 221 478-87396

^b^ Institute for Interdisciplinary Dermatological Prevention and Rehabilitation (iDerm) at the University of Osnabrueck, Am Finkenhuegel 7a, 49076 Osnabrueck, Germany; julia.meyer@uos.de; +49 541 969-7407

^c^ Institute of Human Genetics, University Hospital Cologne, Cologne, Germany

^d^ Department of Neurology, University Hospital Cologne, Cologne, Germany; Cognitive Neuroscience, Institute of Neuroscience and Medicine (INM-3), Research Center Jülich, Jülich, Germany.

^e^ Department of Neurology, Helios University Hospital Wuppertal, Heusnerstr. 40, 42283 Wuppertal, Germany

^f^Department of Geriatrics, St. Franziskus Hospital Lohne Lohne, Germany.

^g^ Laboratory Services Laborarztpraxis Osnabrück Osnabrück, Germany.

^h^ Department of Neurology, University Hospital Cologne, Kerpener Str. 62, 50937 Cologne, Germany; josef.kessler@uk-koeln.de; +49 221 478-4011

^1^Both authors contributed equally.

**Table 1: Predictor analyses of CPT’s cognitive training success**

*Backwards Multiple Regression Predicting Cognitive Improvement of the Cognitive Training with Additional Physical Activity*

|  | Improvement in Verbal short-term memory | |
| --- | --- | --- |
| Predictor | Δ*R*² | β |
| Step1  Baseline VSTM  Education | .24 | -.32  -.25 |
| Step 2  Baseline VSTM | -.05 | -.44^+^ |
| Total *R*²  n | .19  18 |  |

|  | Improvement in Working Memory | |
| --- | --- | --- |
| Predictor | Δ*R*² | β |
| Step 1  Education  IGF-1 | .42* | -.41  -.34 |
| Step 2  Education | -.03 | -.59* |
| Total *R*²  n | .34  18 |  |
|  | Improvement in Verbal Fluency | |
| Predictor | Δ*R*² | β |
| Step 1  Age  Baseline VF  IGF-1 | .52* | .26  -.41  .35 |
| Step 2  Baseline VF  IGF-1 | -.06 | -.49*  .35 |
| Step 3  Baseline VF | -.11 | -.59** |
| Total *R*²  n | .35**  18 |  |
|  | Improvement in Alternating Letter Verbal Fluency | |
| Predictor | Δ*R*² | β |
| Step 1  BDNF  Baseline ALVF  IGF-1  VEGF | .54** | -.4  -.38  .31  -.25 |
| Step 2  BDNF  IGF-1  Baseline ALVF | -.06 | -.35  .36  -.40* |
| Total *R*²  n | .51**  18 |  |
|  | Improvement in Attention | |
| Predictor | Δ*R*² | β |
| Step 1  Baseline A  Physical Stage | .34* | -.60*  -.07 |
| Step 2  Baseline A | -.003 | -.64** |
| Total *R*²  n | .38**  18 |  |

*Note.* A = Attention.IGF-1 = . FM = Figural Memory. BDNF = brain-derived neurotropic factor. WM = working memory. VSTM = verbal short-term memory.

**p* ≤ .05. ***p* ≤ .01 *** *p* ≤ .001.
